# Nocturnal blood pressure dip and parapapillary choroidal microvasculature dropout in normal-tension glaucoma

**DOI:** 10.1038/s41598-020-80705-3

**Published:** 2021-01-08

**Authors:** Joong Won Shin, Youn Hye Jo, Min Kyung Song, Hun Jae Won, Michael S. Kook

**Affiliations:** 1 Department of Ophthalmology, College of Medicine, University of Ulsan, Asan Medical Center, 388-1 Pungnap-2-dong, Songpa-gu, Seoul, 138-736 Korea; 2 Department of Ophthalmology, Konkuk University Hospital, Seoul, Korea

**Keywords:** Glaucoma, Eye diseases

## Abstract

Choroidal microvasculature dropout (CMvD) implies compromised optic nerve head perfusion in glaucoma patients. However, there are conflicting findings whether office-hour systemic blood pressure (BP) is related to the presence of CMvD. The present study investigated which systemic BP parameters, derived from 24-h ambulatory BP monitoring (ABPM), are associated with CMvD as assessed by optical coherence tomography angiography (OCT-A) in normal-tension glaucoma (NTG). This study included 88 eyes of 88 NTG patients who underwent 24-h ABPM and OCT-A imaging. Various systemic BP parameters associated with the presence of CMvD were evaluated using logistic regression analyses. CMvD was detected in 38 NTG eyes (43.2%). NTG eyes with CMvD had nighttime diastolic BP (DBP) dip of greater magnitude and longer duration than eyes without CMvD. In multivariate logistic regression, worse VF mean deviation (MD) (odds ratio [OR] 0.786; *P* = 0.001), greater nighttime DBP dip “%” (OR 1.051; *P* = 0.034), and higher daytime peak IOP (OR 1.459; *P* = 0.013) were significantly associated with the presence of CMvD. Based on our findings that the eyes with CMvD are closely associated with having nighttime DBP dip, NTG patients with CMvD should be recommended to undergo 24-h ABPM.

## Introduction

The role of systemic hypotension in glaucoma has been extensively explored in various population-based as well as clinical studies. Low systemic blood pressure (BP) and reduced ocular perfusion pressure (OPP) were identified as important risk factors for the development and progression of open-angle glaucoma (OAG)^1–9^. Studies including our recent reports have also shown that nocturnal hypotension, particularly its duration and magnitude as determined by 24-h ambulatory BP monitoring (ABPM), significantly impacts on future visual field (VF) progression in patients with normal-tension glaucoma (NTG)^5–11^. Moreover, continuous BP monitoring over 24 h may better reveal circulatory insufficiency related to systemic hypotension than snapshot readings of daytime BP in these patients^7–9^. Thus, 24-h ABPM may facilitate understanding of glaucoma pathogenesis related to systemic hypotension.

The recent advent of optical coherence tomography angiography (OCT-A) has allowed objective and reproducible visualization and measurement of the retinal and choroidal microvasculature. OCT-A has shown localized parapapillary choroidal perfusion impairment, such as choroidal microvasculature dropout (CMvD), in glaucomatous eyes^12,13^. To date, CMvD has been detected more frequently in glaucomatous eyes with central VF defects and advanced VF damage at initial presentation^13–16^, progressive retinal nerve fibre layer (RNFL) and/or VF loss^17,18^, and lower choroid thickness^13^. The presence of CMvD may imply compromised optic nerve head (ONH) perfusion in glaucoma patients, since choroidal circulation within the parapapillary area provides microvascular networks that supply deep ONH structures, such as the lamina and prelaminar tissue^19,20^. While a few studies have reported that CMvD is associated with abnormal BP parameters, such as low diastolic BP (DBP), OPP, or mean arterial pressure (MAP)^13,15^, other studies have not confirmed its association with systemic hypotension in glaucoma patients^16,21^. These conflicting findings may stem from the use of snapshot daytime BP measurements, which may not accurately reflect the true nature of the BP abnormality or its circadian rhythm as related to glaucoma pathogenesis.

Given that nocturnal hypotension is a known systemic vascular risk factor for glaucoma^7–9^, we hypothesized that a nighttime BP dip may be closely linked to CMvD, since it can give rise to a localized choroidal perfusion defect in the form of microvasculature dropout, and these conditions may share circulatory insufficiency to the ONH in common. Therefore, we here investigated the relationship of various 24-h BP parameters, including nocturnal BP dip parameters as determined utilizing 24-h ABPM, with the presence of CMvD in NTG patients. The clarification of this relationship could provide important insights into the role of CMvD and vascular insufficiency in the pathophysiology of NTG.

## Results

Of the 95 eyes of 95 NTG patients who initially met the inclusion criteria, 7 eyes were excluded because of poor quality OCT-A images. Eighty-eight eyes of 88 NTG patients (35 men and 53 women; mean age of 56.0 ± 12.2 years) were included in the final analysis. Of these patients, 19 (21.6%) had a history of systemic hypertension and were taking oral antihypertensive medication. In subgroup analysis using eyes with and without CMvD, matched by VF mean deviation (MD; ≤ 1 dB) and age (≤ 10 years), 30 eyes were assigned to each subgroup, with a mean age of 57.1 ± 12.2 years. There were excellent interobserver agreement in terms of determining the presence of CMvD (k = 0.904; 95% confidence interval [CI] 0.832–0.975, *P* < 0.001). The interobserver intraclass correlation coefficient (ICC) for β-zone parapapillary atrophy (β-PPA) area measurement was 0.944 (95% CI 0.908–0.979).

CMvD was identified in 38 (43.2%) of 88 eyes of NTG patients. Table 1 summarizes the demographic, ocular, and systemic characteristics of eyes with and without CMvD. Eyes with CMvD had significantly worse VF MD, worse VF pattern standard deviation (PSD), lower average circumpapillary vessel density (cpVD), lower circumpapillary RNFL thickness (cpRNFLT), and larger β-PPA area than eyes without CMvD (all *P* < 0.05). There were no significant differences between the 2 groups with respect to age, sex, refractive error (RE), axial length (AL), central corneal thickness (CCT), untreated office intraocular pressure (IOP), office systolic BP (SBP), DBP, the presence of optic disc hemorrhage, number of patients with hypertension and antihypertensive medication use, diabetes mellitus, stroke, migraine, and cold extremities. In subgroup analysis using eyes with and without CMvD, matched for glaucoma severity and age, eyes with CMvD showed more myopic RE and AL measurements (*P* = 0.028 and 0.047) than eyes without CMvD, whereas VF MD, PSD, cpVD, cpRNFLT, and β-PPA area did not differ between the 2 subgroups. Other demographic, ocular, and systemic characteristics did not differ between the 2 subgroups (Table 1).

**Table 1:** Clinical characteristics of eyes with and without choroidal microvasculature dropout (CMvD) in normal-tension glaucoma.

	All subjects (n = 88)			MD- and age-matched subjects (n = 60)		
	Eyes with CMvD (n = 38)	Eyes without CMvD (n = 50)	P	Eyes with CMvD (n = 30)	Eyes without CMvD (n = 30)	P
**Demographic characteristics**						
Age	55.5 ± 11.5	56.3 ± 12.8	0.756	54.5 ± 11.5	59.7 ± 12.6	0.100
Sex	12/26	23/27	0.171	11/19	12/18	0.791
**Ocular characteristics**						
SE	− 2.44 ± 3.00	− 1.95 ± 3.13	0.468	− 2.91 ± 3.0	− 1.28 ± 2.6	**0.028**
AL	25.00 ± 1.43	24.54 ± 1.48	0.161	25.17 ± 1.44	24.35 ± 1.57	**0.047**
CCT	535.0 ± 33.8	540.8 ± 32.5	0.421	539.5 ± 35.1	533.3 ± 32.8	0.487
Untreated office IOP	14.1 ± 1.8	13.5 ± 1.8	0.088	14.2 ± 1.8	13.6 ± 1.9	0.166
Optic disc hemorrhage	6/32	5/45	0.416	1/29	4/26	0.161
VF MD	− 7.83 ± 6.10	− 3.27 ± 3.35	** < 0.001**	− 5.28 ± 3.42	− 4.96 ± 3.15	0.705
VF PSD	9.63 ± 4.61	5.39 ± 3.15	** < 0.001**	8.45 ± 4.45	6.52 ± 3.41	0.065
cpVD	41.6 ± 6.0	46.0 ± 5.3	** < 0.001**	43.0 ± 4.8	44.9 ± 5.4	0.181
cpRNFLT	72.1 ± 11.6	77.2 ± 9.9	**0.031**	75.0 ± 10.5	76.2 ± 9.9	0.650
β-PPA area	1.11 ± 0.29	0.95 ± 0.34	**0.019**	1.09 ± 0.26	0.94 ± 0.36	0.084
**Systemic characteristics**						
Office systolic blood pressure	126.1 ± 16.6	118.9 ± 17.6	0.056	125.6 ± 17.1	117.5 ± 15.1	0.055
Office diastolic blood pressure	73.6 ± 10.1	77.9 ± 11.1	0.068	73.8 ± 10.7	78.7 ± 12.2	0.107
Hypertension	9/29	10/40	0.677	6/24	7/23	0.754
Diabetes mellitus	6/32	4/46	0.254	4/26	3/27	0.688
Migraine	5/33	3/47	0.437	3/27	2/28	0.640
Cold extremity	4/34	3/47	0.247	4/26	2/28	0.389

Statistically significant differences are shown in bold.

*MD* mean deviation, *SE* spherical equivalent, *AL* axial length, *CCT* central corneal thickness, *IOP* intraocular pressure, *VF* visual field, *PSD* pattern standard deviation, *VD* vessel density, *cpVD* circumpapillary vessel density, *cpRNFLT* circumpapillary retinal nerve fibre layer thickness, *β-PPA* beta parapapillary atrophy.

**Figure 1:**
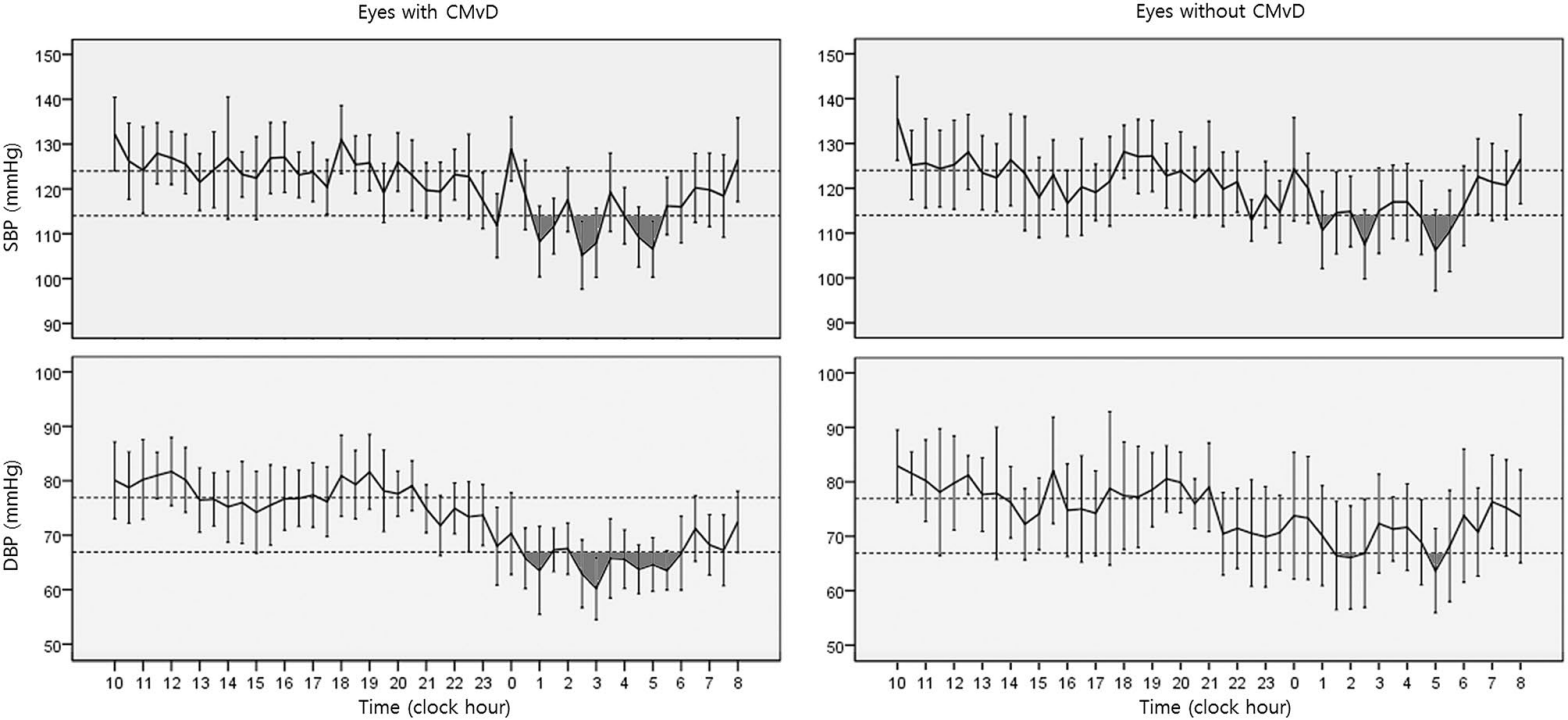
Overall 24-h blood pressure (BP) patterns of eyes with and without choroidal microvasculature dropout (CMvD) in the entire patient group (n = 88). Nighttime diastolic BP (DBP) in eyes with CMvD showed a longer duration and greater magnitude of BP dip (black area; at least 10 mmHg below the mean daytime BP) than those in eyes without CMvD. However, nighttime systolic BP (SBP) did not show differences in patterns of duration and magnitude of BP dip between eyes with and without CMvD.

Table 2 shows in-hospital 24-h daytime and nighttime BP and IOP measurements in eyes with and without CMvD. In all subjects, daytime and nighttime SBP parameters did not differ significantly between eyes with and without CMvD (all *P* > 0.05). Nighttime DBP dip “%” (19.7% vs. 12.8%, *P* = 0.009), dip “time” (1.7 h and 1.1 h, *P* = 0.041), and dip “area” (16.1 mmHg h vs. 8.7 mmHg h, *P* = 0.037) were significantly greater in eyes with CMvD that in those without CMvD, whereas other daytime and nighttime DBP parameters did not show significant differences between the 2 groups (all *P* > 0.05). Among 24-h IOP parameters, eyes with CMvD showed significantly higher daytime peak IOP than eyes without CMvD. Figure 1 shows the overall 24-h BP patterns of eyes with and without CMvD in the entire patient group. Nighttime DBP in eyes with CMvD showed a BP dip with a longer duration and greater magnitude (i.e., at least 10 mmHg below mean daytime BP) than those in eyes without CMvD. However, nighttime SBP did not differ in patterns of duration and magnitude of BP dip between eyes with and without CMvD.

**Table 2:** Comparison of in-hospital 24-h blood pressure and intraocular pressure between eyes with and without choroidal microvasculature dropout (CMvD) in eyes with normal-tension glaucoma.

	All subjects (n = 88)			MD- and age-matched subjects (n = 60)		
	Eyes with CMvD (n = 38)	Eyes without CMvD (n = 50)	P	Eyes with CMvD (n = 30)	Eyes without CMvD (n = 30)	P
**Systolic blood pressure**						
Daytime mean	125.5 ± 12.6	124.0 ± 13.4	0.615	125.1 ± 11.9	122.7 ± 13.3	0.458
Daytime peak	145.6 ± 21.1	143.6 ± 20.9	0.664	142.6 ± 17.0	140.6 ± 20.3	0.681
Daytime trough	108.9 ± 12.4	106.3 ± 11.9	0.330	109.3 ± 11.3	106.1 ± 12.4	0.300
Daytime range	36.7 ± 15.2	37.2 ± 17.4	0.870	33.3 ± 13.0	34.5 ± 17.3	0.763
Daytime dip '%'	13.2 ± 4.9	14.1 ± 5.8	0.432	12.6 ± 4.7	13.5 ± 5.4	0.509
Daytime dip “time” (h)	2.7 ± 1.8	2.6 ± 1.5	0.737	2.5 ± 1.8	2.3 ± 1.3	0.670
Daytime dip “area” (mmHg h)	15.5 ± 15.1	22.8 ± 38.1	0.276	14.0 ± 15.6	16.0 ± 18.3	0.643
Nighttime mean	117.2 ± 12.9	118.2 ± 15.8	0.753	117.7 ± 13.6	118.0 ± 14.7	0.945
Nighttime peak	129.7 ± 15.2	128.2 ± 20.2	0.719	129.9 ± 14.8	126.3 ± 16.8	0.391
Nighttime trough	104.2 ± 13.6	107.6 ± 15.3	0.270	105.2 ± 14.8	109.3 ± 15.4	0.297
Nighttime range	25.5 ± 13.7	20.6 ± 15.4	0.126	24.7 ± 11.7	17.1 ± 10.7	**0.011**
Nighttime dip '%'	16.7 ± 9.7	13.1 ± 9.8	0.088	15.9 ± 9.1	10.9 ± 8.7	**0.033**
Nighttime dip “time” (h)	2.4 ± 1.5	2.1 ± 2.1	0.450	2.3 ± 1.5	1.9 ± 2.1	0.403
Nighttime dip “area” (mmHg h)	29.1 ± 29.6	22.7 ± 29.5	0.326	26.4 ± 27.4	16.8 ± 23.2	0.157
**Diastolic blood pressure**						
Daytime mean	78.1 ± 7.2	76.9 ± 8.2	0.469	77.6 ± 7.3	75.8 ± 9.1	0.417
Daytime peak	94.2 ± 15.7	90.6 ± 13.4	0.256	92.3 ± 15.2	87.4 ± 10.4	0.157
Daytime trough	64.1 ± 13.8	64.2 ± 9.5	0.959	64.8 ± 14.4	64.3 ± 9.7	0.892
Daytime range	30.1 ± 18.8	26.4 ± 13.6	0.288	27.5 ± 19.1	23.1 ± 8.5	0.253
Daytime dip '%'	18.3 ± 15.5	16.7 ± 7.4	0.513	16.9 ± 16.1	15.4 ± 5.8	0.620
Daytime dip “time” (h)	1.4 ± 1.7	1.8 ± 1.9	0.310	1.3 ± 1.8	1.6 ± 1.6	0.516
Daytime dip “area” (mmHg h)	10.4 ± 18.9	11.6 ± 23.6	0.788	8.9 ± 19.3	5.3 ± 7.5	0.345
Nighttime mean	72.4 ± 8.2	74.8 ± 10.1	0.234	72.4 ± 8.7	75.6 ± 10.5	0.199
Nighttime peak	80.8 ± 8.8	82.6 ± 12.5	0.441	80.7 ± 8.7	82.1 ± 12.1	0.610
Nighttime trough	62.6 ± 10.2	66.9 ± 10.7	0.056	62.9 ± 10.7	69.2 ± 10.2	**0.023**
Nighttime range	18.2 ± 9.8	15.7 ± 11.9	0.286	17.8 ± 9.5	12.9 ± 8.0	**0.034**
Nighttime dip '%'	19.7 ± 12.2	12.8 ± 11.9	**0.009**	18.8 ± 12.4	8.6 ± 9.4	**0.001**
Nighttime dip “time” (h)	1.7 ± 1.3	1.1 ± 1.6	**0.041**	1.7 ± 1.4	0.5 ± 0.9	** < 0.001**
Nighttime dip “area” (mmHg h)	16.1 ± 18.3	8.7 ± 14.2	**0.037**	15.3 ± 18.5	2.6 ± 5.8	**0.001**
**Intraocular pressure**						
Daytime mean (sitting)	14.0 ± 1.8	13.3 ± 1.8	0.071	14.2 ± 1.8	13.4 ± 1.9	0.118
Daytime peak (sitting)	16.7 ± 2.4	15.7 ± 2.1	**0.045**	16.8 ± 2.3	15.7 ± 2.2	0.059
Daytime range (sitting)	5.1 ± 1.9	4.5 ± 1.1	0.078	5.1 ± 1.9	4.3 ± 1.0	**0.048**
Nighttime mean (supine)	14.4 ± 2.2	13.7 ± 2.3	0.195	14.3 ± 2.2	13.8 ± 2.1	0.362
Nighttime peak (supine)	16.5 ± 2.4	15.9 ± 2.4	0.267	16.5 ± 2.3	16.0 ± 2.2	0.461
Nighttime range (supine)	4.1 ± 1.7	4.3 ± 1.6	0.723	4.2 ± 1.8	4.3 ± 1.7	0.826

Statistically significant differences are shown in bold.

*MD* mean deviation.

In VF MD- and age-matched subgroup analyses (Table 2), nighttime SBP range (24.7 mmHg vs. 17.1 mmHg, *P* = 0.011) and dip “%” (15.9% vs. 10.9%, *P* = 0.033), nighttime DBP trough (62.9 mmHg vs. 69.2 mmHg, *P* = 0.023), range (17.8 mmHg vs. 12.9 mmHg, *P* = 0.034), dip “%” (18.8% vs. 8.6%, *P* = 0.001), dip “time” (1.7 h vs. 0.5 h, *P* < 0.001), and dip “area” (15.3 mmHg h vs. 2.6 mmHg h, *P* = 0.001) were significantly greater in eyes with CMvD that in those without CMvD. Daytime SBP and DBP parameters did not show differences between the 2 subgroups (all *P* > 0.05). Eyes with CMvD showed a wider daytime IOP range than eyes without CMvD in subgroup analysis (*P* = 0.048).

The factors associated with the presence of CMvD were determined using logistic regression analyses (Tables 3, 4, and 5). In all subjects, worse VF MD, lower cpVD, lower cpRNFLT, larger β-PPA area, greater nighttime DBP dip (“%”, “time”, and “area”), and higher daytime peak IOP were significantly associated with the presence of CMvD, in univariate analyses (all *P* < 0.05, Table 3). In multivariate analyses, worse VF MD (odds ratio [OR], 0.786; *P* = 0.001), greater nighttime DBP dip “%” (OR 1.051; *P* = 0.034), and higher daytime peak IOP (OR 1.459; *P* = 0.013) were significantly associated with the presence of CMvD (Table 4). In VF MD- and age-matched subgroup analyses, greater nighttime SBP range and dip “%”, lower nighttime DBP trough, and greater nighttime DBP range and dip (“%”, “time”, and “area”) were significantly associated with the presence of CMvD in univariate analyses (*P* < 0.05, Table 3). In multivariate analyses with the subgroup, greater nighttime DBP dip “time” (OR 4.418; *P* = 0.031) and “area” (OR 1.182; *P* = 0.048) showed significant associations with the presence of CMvD (Table 5).

**Table 3:** Results of univariate logistic regression analysis for determining the factors associated with the presence of choroidal microvasculature dropout in eyes with normal-tension glaucoma.

	Univariate model 1 (n = 88, all subjects)			Univariate model 2 (n = 60, MD- and age- matched subjects)		
	OR	95% CI	P	OR	95% CI	P
Age	0.994	0.960–1.030	0.753	0.964	0.922–1.008	0.103
Sex	0.542	0.224–1.308	0.173	0.868	0.306–2.461	0.791
CCT	0.995	0.982–1.008	0.417	1.006	0.990–1.021	0.480
AL	1.245	0.916–1.691	0.161	1.448	0.996–2.104	0.052
Hypertension	1.241	0.448–3441	0.678	1.217	0.355–4.170	0.754
Diabetes	2.156	0.563–8.261	0.262	1.385	0.282–6.796	0.688
Migraine	2.374	0.530–10.627	0.258	1.556	0.241–10.049	0.643
Cold extremity	1.843	0.387–8.777	0.443	2.154	0.363–12.764	0.398
Optic disc hemorrhage	1.687	0.474–6.011	0.420	4.462	0.468–42.514	0.194
Untreated office IOP	1.229	0.968–1.561	0.120	1.224	0.920–1.628	0.165
VF MD	0.804	0.713–0.906	** < 0.001**	0.970	0.829–1.134	0.699
cpVD	0.868	0.796–0.946	**0.001**	0.931	0.838–1.034	0.183
cpRNFLT	0.956	0.916–0.997	**0.036**	0.988	0.939–1.039	0.644
β-PPA area	5.133	1.264–20.840	**0.022**	4.366	0.806–23.643	0.087
Office systolic blood pressure	1.025	0.999–1.052	0.062	1.034	0.999–1.071	0.056
Office diastolic blood pressure	0.961	0.920–1.004	0.074	0.960	0.918–1.005	0.080
Office mean arterial pressure	0.997	0.958–1.037	0.869	0.997	0.952–1.045	0.907
**Systolic blood pressure**						
Daytime mean	1.009	0.976–1.042	0.610	1.016	0.975–1.059	0.452
Daytime peak	1.005	0.984–1.025	0.660	1.006	0.979–1.034	0.675
Daytime trough	1.018	0.982–1.055	0.327	1.024	0.979–1.071	0.297
Daytime range	0.998	0.972–1.024	0.868	0.995	0.962–1.029	0.758
Daytime dip “%”	0.968	0.893–1.049	0.428	0.965	0.871–1.070	0.503
Daytime dip “time” (h)	1.047	0.802–1.367	0.734	1.075	0.775–1.490	0.665
Daytime dip “area” (mmHg h)	0.989	0.968–1.010	0.309	0.993	0.962–1.024	0.638
Nighttime mean	0.995	0.967–1.025	0.749	0.999	0.963–1.036	0.943
Nighttime peak	1.004	0.981–1.028	0.715	1.015	0.982–1.049	0.386
Nighttime trough	0.983	0.955–1.013	0.268	0.982	0.948–1.016	0.293
Nighttime range	1.023	0.993–1.055	0.134	1.065	1.012–1.121	**0.016**
Nighttime dip “%”	1.039	0.994–1.086	0.102	1.067	1.003–1.135	**0.039**
Nighttime dip “time” (h)	1.094	0.868–1.379	0.446	1.130	0.852–1.498	0.397
Nighttime dip “area” (mmHg h)	1.007	0.993–1.022	0.324	1.015	0.994–1.037	0.159
**Diastolic blood pressure**						
Daytime mean	1.021	0.966–1.079	0.465	1.027	0.964–1.094	0.411
Daytime peak	1.017	0.987–1.048	0.259	1.031	0.987–1.077	0.166
Daytime trough	0.999	0.963–1.037	0.959	1.003	0.962–1.046	0.890
Daytime range	1.015	0.988–1.042	0.288	1.022	0.984–1.061	0.259
Daytime dip “%”	1.012	0.976–1.051	0.513	1.011	0.967–1.058	0.619
Daytime dip “time” (h)	0.880	0.688–1.125	0.308	0.904	0.670–1.220	0.509
Daytime dip “area” (mmHg h)	0.997	0.977–1.018	0.786	1.019	0.979–1.061	0.358
Nighttime mean	0.972	0.927–1.019	0.234	0.965	0.913–1.019	0.200
Nighttime peak	0.984	0.946–1.024	0.438	0.987	0.940–1.037	0.604
Nighttime trough	0.960	0.920–1.002	0.060	0.942	0.892–0.994	**0.030**
Nighttime range	1.021	0.982–1.062	0.286	1.068	1.003–1.138	**0.040**
Nighttime dip “%”	1.049	1.011–1.090	**0.012**	1.097	1.032–1.167	**0.003**
Nighttime dip “time” (h)	1.351	1.006–1.814	**0.046**	2.587	1.522–4.397	** < 0.001**
Nighttime dip “area” (mmHg h)	1.029	1.001–1.058	**0.044**	1.120	1.033–1.215	**0.006**
**Intraocular pressure**						
Daytime mean (sitting)	1.249	0.978–1.595	0.104	1.254	0.943–1.670	0.120
Daytime peak (sitting)	1.224	1.001–1.497	**0.049**	1.249	0.987–1.581	0.064
Daytime range (sitting)	1.295	0.966–1.736	0.084	1.436	0.988–2.089	0.058
Nighttime mean (supine)	1.135	0.937–1.376	0.195	1.121	0.880–1.428	0.356
Nighttime peak (supine)	1.107	0.926–1.322	0.266	1.092	0.867–1.374	0.455
Nighttime range (supine)	0.954	0.739–1.232	0.719	0.967	0.720–1.297	0.822

Statistically significant differences are shown in bold.

*MD* mean deviation, *CCT* central corneal thickness, *AL* axial length, *IOP* intraocular pressure, *VF* visual field, *cpVD* circumpapillary vessel density, *cpRNFLT* circumpapillary retinal nerve fibre layer thickness, *β-PPA* beta parapapillary atrophy.

**Table 4:** Results of multivariate logistic regression analysis for determining the factors associated with presence of choroidal microvasculature dropout in all study subjects.

	Multivariate model (n = 88, all subjects)			
	OR	95% CI	VIF**	P
VF MD	0.786	0.681–0.906	1.914	**0.001**
cpVD	Dropped		3.748	
cpRNFLT	Dropped		2.634	
β-PPA area	6.282	0.910–43.369	1.179	0.062
Office SBP	Dropped		1.413	
Office DBP	Dropped		1.531	
Nighttime DBP trough	Dropped		3.474	
Nighttime DBP dip “%”	1.051	1.004–1.101	5.417	**0.034**
Nighttime DBP dip “time” (h)	Dropped		2.376	
Nighttime DBP dip “area” (mmHg h)	Dropped		4.969	
Daytime IOP peak (sitting)	1.459	1.084–1.964	1.415	**0.013**
Daytime IOP range (sitting)	Dropped		1.704	

A backward elimination process was used to build a multivariate logistic regression model incorporating variables with P < 0.10 in univariate analysis. Statistically significant differences are shown in bold.

*OR* odds ratio, *CI* confidence interval, *VIF* variance inflation factor, *VF* visual field, *MD* mean deviation, *cpVD* circumpapillary vessel density, *cpRNFLT* circumpapillary retinal nerve fibre layer thickness, *β-PPA* beta parapapillary atrophy, *SBP* systolic blood pressure, *DBP* diastolic blood pressure, *IOP* intraocular pressure.

**Table 5:** Results of multivariate logistic regression analysis for determining the factors associated with the presence of choroidal microvasculature dropout in visual field mean deviation- and age-matched subjects.

	Multivariate model (n = 60, MD- and age-matched subjects)			
	OR	95% CI	VIF**	P
AL	Dropped		1.346	
β-PPA area	7.721	0.759–78.502	1.375	0.084
Office SBP	Dropped		2.063	
Office DBP	0.945	0.878–1.017	1.876	0.131
Nighttime SBP range	Dropped		3.851	
Nighttime SBP dip “%”	0.864	0.742–1.006	3.739	0.060
Nighttime DBP trough	Dropped		2.915	
Nighttime DBP range	0.837	0.696–1.008	3.119	0.060
Nighttime DBP dip “%”	Dropped		6.665	
Nighttime DBP dip “time” (h)	4.418	1.144–17.057	2.596	**0.031**
Nighttime DBP dip “area” (mmHg h)	1.182	1.001–1.394	6.190	**0.048**
Daytime IOP peak (sitting)	1.303	0.910–1.865	1.811	0.148
Daytime IOP range (sitting)	Dropped		2.344	

A backward elimination process was used to build the multivariate logistic regression model incorporating variables with P < 0.10 in univariate analysis. Statistically significant differences are shown in bold.

*MD* mean deviation, *OR* odds ratio, *CI* confidence interval, *VIF* variance inflation factor, *AL* axial length, *β-PPA* beta parapapillary atrophy, *SBP* systolic blood pressure, *DBP* diastolic blood pressure, *IOP* intraocular pressure.

Table 6 shows the results of logistic regression analyses for determining clinical factors associated with the presence of extreme nighttime DBP dip. In all subjects, the presence of CMvD and lower office-hour DBP were associated with the presence of extreme nighttime DBP dip, in univariate analyses (*P* < 0.05). In VF MD- and age-matched subgroups, the presence of CMvD was associated with the presence of an extreme nighttime DBP dip, in univariate analyses (*P* < 0.05). Multivariate analyses revealed that the presence of CMvD was a significant factor associated with the presence of an extreme nighttime DBP dip, after adjusting for office-hour DBP in all subjects (OR 2.488; *P* = 0.047) and in the VF MD- and age-matched subgroup (OR 7.087; *P* = 0.019).

**Table 6:** Factors associated with extreme nighttime diastolic blood pressure dip using logistic regression analyses.

	Univariate model (n = 88, all subjects)			Multivariate model^a^ (n = 88, all subjects)			Univariate model (n = 60, MD- and age-matched subjects)			Multivariate model^a^ (n = 60, MD and age-matched subjects)		
	OR	95% CI	P	OR	95% CI	P	OR	95% CI	P	OR	95% CI	P
Age	0.996	0.961–1.031	0.806				1.002	0.952–1.054	0.944			
Sex	0.946	0.399–2.245	0.900				0.993	0.281–3.510	0.991			
CCT	1.001	0.988–1.014	0.864				0.999	0.980–1.017	0.887			
AL	0.930	0.689–1.257	0.638				1.130	0.757–1.687	0.550			
Hypertension	1.003	0.359–2.806	0.995				1.039	0.188–5.729	0.965			
Diabetes	1.437	0.384–5.377	0.590				1.756	0.192–16.049	0.618			
Migraine	2.500	0.558–11.200	0.231				1.116	0.114–10.944	0.925			
Cold extremity	1.902	0.348–10.389	0.458				1.381	0.400–4.767	0.610			
Optic disc hemorrhage	1.312	0.355–4.858	0.684				1.116	0.114–10.944	0.925			
Untreated office IOP	0.918	0.728–1.158	0.918				0.997	0.715–1.392	0.988			
VF MD	0.960	0.884–1.041	0.323				0.904	0.750–1.090	0.289			
cpVD	0.955	0.888–1.026	0.208				0.956	0.851–1.075	0.453			
Presence of CMvD	2.625	1.097–6.284	**0.030**	2.488	1.013–6.112	**0.047**	8.105	1.612–40.766	**0.011**	7.087	1.383–36.321	**0.019**
cpRNFLT	0.984	0.946–1.023	0.418				1.051	0.985–1.121	0.130			
β-PPA area	3.175	0.816–12.355	0.106				2.513	0.337–18.722	0.368			
Office SBP	1.025	0.997–1.055	0.125				1.026	0.987–1.066	0.193			
Office DBP	0.944	0.899–0.992	**0.022**	0.946	0.900–0.996	**0.033**	0.888	0.888–1.010	0.096	0.957	0.894–1.025	0.213

*MD* mean deviation, *OR* odds ratio, *CI* confidence interval, *CCT* central corneal thickness, *AL* axial length, *IOP* intraocular pressure, *VF* visual field, *cpVD* circumpapillary vessel density, *CMvD* choroidal microvasculature dropout, *cpRNFLT* circumpapillary retinal nerve fibre layer thickness, *β-PPA* beta parapapillary atrophy, *SBP* systolic blood pressure, *DBP* diastolic blood pressure.

^a^A backward elimination process was used to build a multivariate logistic regression model incorporating variables with P < 0.10 in univariate analysis. Statistically significant differences are shown in bold.

**Figure 2:**
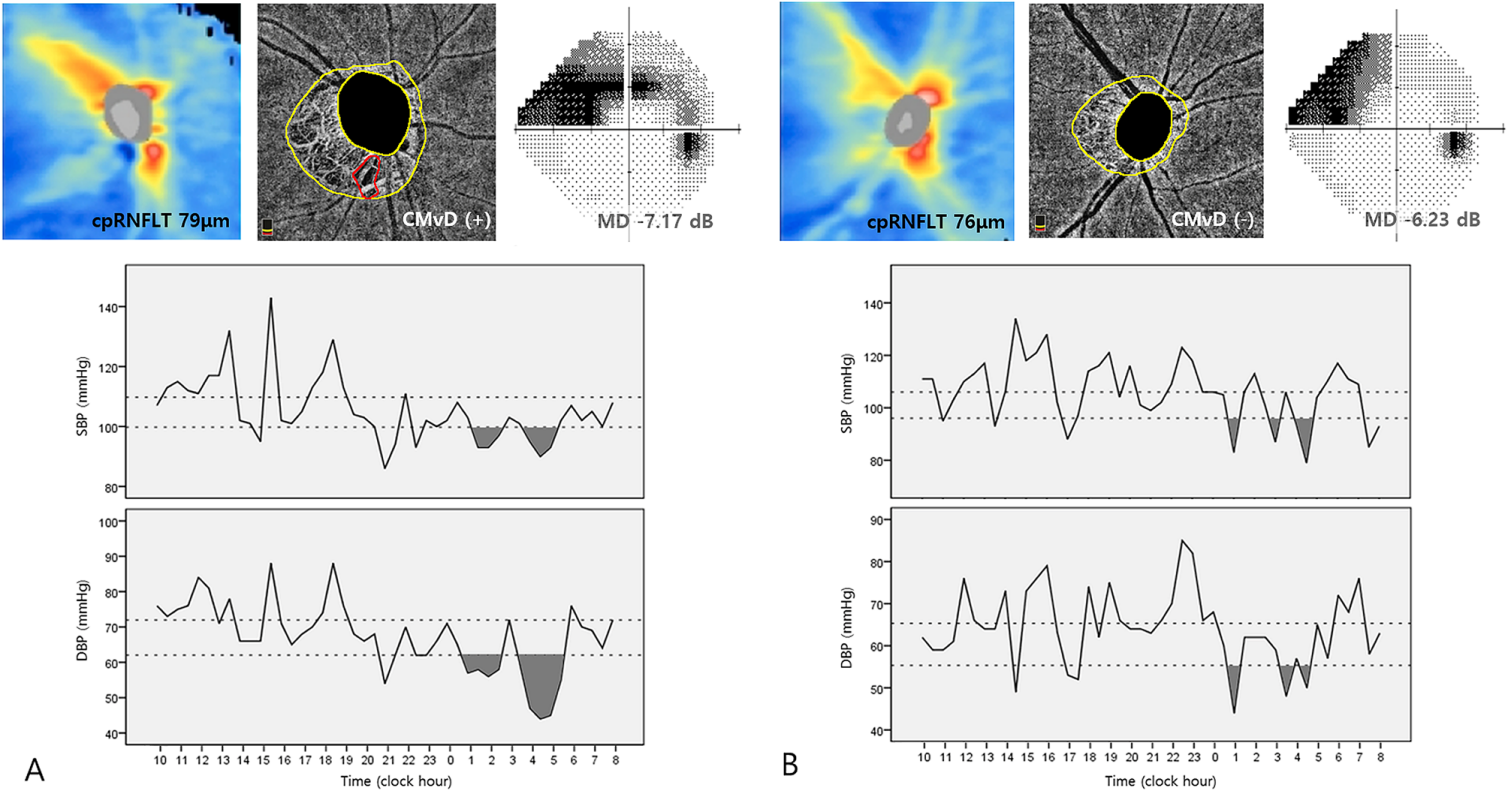
Representative cases of normal-tension glaucoma eyes with and without choroidal microvasculature dropout (CMvD). A 52-year-old woman with CMvD as indicated by red outline in the β-zone parapapillary atrophy shown by yellow outlines (**A**) and a 56-year-old woman without CMvD (**B**) had similar circumpapillary retinal nerve fibre layer loss (thickness, 79 μm vs. 76 μm) in the inferotemporal area and visual field defect (mean deviation, − 7.17 dB vs. − 6.23 dB, respectively) in the superior hemifield. Nevertheless, 24-h blood pressure (BP) patterns showed differences depending on the presence of CMvD. Nighttime diastolic BP (DBP) in the eye with CMvD showed a longer duration and greater magnitude of BP dip (black area; at least 10 mmHg below the mean daytime BP) than those in the eye without CMvD. However, nighttime systolic BP (SBP) showed similar patterns of BP dip in eyes with and without CMvD.

Representative cases are shown in Fig. 2. A 52-year-old woman with CMvD as indicated by red arrow in the β-PPA (Fig. 2A) and 56-year-old woman without CMvD (Fig. 2B) had similar severity of glaucomatous damage (VF MD − 7.17 dB vs. − 6.23 dB, respectively; cpRNFLT, 79 μm vs. 76 μm, respectively). Nevertheless, 24-h BP patterns showed differences, depending on the presence of CMvD. Nighttime DBP in the eye with CMvD showed a longer duration and greater magnitude of BP dip (i.e., at least 10 mmHg below the mean daytime BP) than those in the eye without CMvD. However, nighttime SBP showed similar patterns of BP dips in eyes with and without CMvD.

## Discussion

The presence of CMvD has been reported to be associated with systemic vascular insufficiency, such as lower office-hour DBP, MAP, and OPP^13,15^, and may represent a regional filling defect of the parapapillary choroid in glaucoma patients^12^. These findings suggest that CMvD may be a sign of ischemia in the parapapillary choroid and ONH, rather than being secondary to glaucomatous damage^13,15^. Since BP is a dynamic biologic parameter, a single snap-shot measurement of BP during office hours may not accurately reflect the true relationship between circadian BP readings and CMvD in glaucomatous patients. Using 24-h ABPM, Hayreh et al.^22^ demonstrated that significant nocturnal DBP dip may reduce the ONH blood flow to below a critical level, and thereby may contribute significantly to the pathogenesis of NTG. In the current study, nighttime DBP dip parameters were significantly associated with the presence of CMvD in the entire group, as well as in subgroup analyses in NTG eyes with and without CMvD. Moreover, the presence of CMvD was also an independent predictor of an extreme nighttime DBP dip in both analyses. Our findings, therefore, suggest that nocturnal hypotension, as expressed in the magnitude and duration of DBP dip (“%”, “area”, and “time”), may be independently linked to CMvD, a parapapillary choroidal perfusion defect. To our knowledge, no previous study has assessed the association between nocturnal BP dip and CMvD detection in NTG eyes using 24-h ABPM data; our findings may provide additional knowledge regarding the clinical implications of CMvD in glaucoma.

Nocturnal hypotension has been regarded as a significant predictor of glaucoma progression^4–9,23^. Charlson et al.^7^ reported that the duration and magnitude of nocturnal MAP dip were associated with progressive VF loss in NTG eyes. Our group recently reported that the nocturnal DBP dip, in terms of its duration and magnitude, had a significant impact on glaucomatous VF progression in NTG eyes^9^. Since DBP has been considered as a major determinant of tissue perfusion in various end organs including eye^9,24–31^, chronic ischemia on the ONH was proposed as a possible mechanism for the relationship between nocturnal hypotension and future glaucoma progression^5,32^. Of note, we found that eyes with CMvD had a significant association with nighttime DBP dip, as expressed by “%”, “time”, and “area” in both the entire group and subgroup analyses, irrespective of differences in the glaucoma severity (Tables 4 and 5). Therefore, it is not surprising that the presence of CMvD may be a significant predictor of future VF and RNFL progression, as reported in previous studies^17,18,33^.

Contrary to previous studies^13,15^, none of the office-hour BP parameters showed significant association with CMvD in both the entire group and the VF MD- and age-matched subgroup analyses in the current study. Since short- and long-term variabilities are important characteristics of systemic BP, different study results can be derived from snapshot BP readings, depending on when BP is measured during office hours. For this reason, 24-h ABPM data is considered a better predictor of end-organ disease, such as glaucoma, than single daytime BP readings^7–9^. In a study of 93 participants from the Maracaibo Aging Study^8^, extreme nocturnal BP dip, defined as a decrease of more than 20% of the nocturnal BP levels relative to daytime BP levels in 24-h ABPM outcomes, was found to be a more useful indicator of glaucomatous damage than once-off measurement of office-hour BP. Consequently, various nighttime and daytime BP dip parameters, derived from 24-h ABPM as outlined in our study, may provide more accurate information regarding the true association between systemic BP dip and CMvD in NTG patients.

Although vascular autoregulation preserves constant blood flow in the ONH against BP instability or fluctuation, this adaptive mechanism cannot compensate for extreme nocturnal BP dip, which can be detected by 24-h ABPM in some patients^4^. This may explain our finding that nighttime DBP dip parameters (“%”, “time”, and “area”) were significantly associated with the presence of CMvD in a multivariate model in both the entire group and subgroup analyses, while office-hour BP did not show any association. Our findings suggest that a large magnitude or long duration of DBP dip at nighttime may reduce the blood flow below a critical level, impairing parapapillary choroid perfusion in the form of CMvD. Therefore, 24-h ABPM may have an advantage in assessing the risk of a regional perfusion defect in the ONH, represented by CMvD. Future prospective and longitudinal studies are needed to elucidate a temporal relationship between nocturnal hypotension and development or enlargement of a CMvD in NTG patients.

Although nocturnal hypotension, particularly a nocturnal DBP dip, is associated with ONH ischemia and progressive VF loss in NTG eyes^9,23^, the association between extreme nighttime DBP dip and CMvD has not been studied. In the current study, the presence of CMvD was an independent predictor of extreme nighttime DBP dip, irrespective of the patient group analysed (Table 6). DBP has been regarded as a major determinant of perfusion status in various end organs, including the eye^9,24–31^. Although it remains to be clarified, an explanation for the association of CMvD with extreme nocturnal DBP dip is that nighttime DBP dip may lead to compromised perfusion in the posterior ciliary arteries (PCAs), which supply both the parapapillary choroid and the deep optic nerve tissues^19,34^. This may, in turn, lead to regional perfusion deficiency, such as CMvD, at the location of the watershed zone, which is vulnerable to ischemic conditions, such as a systemic DBP dip. Fluorescein angiography showed that non-filling of the watershed zone in the human eye is found at the temporal part of ONH and the parapapillary choroid between the lateral and medial PCAs^34^. As such, the temporal parapapillary choroid within the β-PPA is the most common location of CMvD in glaucomatous eyes^14–16^.

IOP has a significant impact on the development and progression of NTG. Crichton et al.^35^ reported that inter-eye differences in IOP do occur in patients with NTG, and that VF damage was greater in the eye with the higher mean IOP. Both daytime and nighttime IOP peaks have been linked to glaucomatous progression^36,37^. In the current study, daytime IOP peak was significantly associated with the presence of CMvD in the multivariate model using the entire patient group, in addition to VF MD and nighttime DBP dip “%” (Table 4). In contrast, previous studies have not found significant association between IOP parameters and the presence of CMvD^13,15^. Although the reasons for this discrepancy remain unclear, it may be related to whether IOP lowering agents were used in the study subjects. Our treatment-naïve patients reflect the actual relationship between IOP and the presence of CMvD, since higher baseline IOP prior to treatment may induce mechanical stress in the vulnerable zone of the ONH, such as the temporal part of the ONH and the parapapillary choroid, independent of ischemic compromise. Interestingly, however, the impact of the daytime IOP peak was no longer significant in the multivariate model of VF MD- and age-matched subgroups (Table 5).

Systemic vascular risk factors, such as systemic hypotension, nocturnal BP dip, and large BP variability, have been reported to be associated with parafoveal scotoma in NTG^38,39^. It remains unclear how parafoveal scotoma is related to eyes with these systemic vascular risk factors. Recent OCT-A studies showed that parafoveal scotoma is related to the presence of CMvD^15,16^. Interestingly, Lee et al.^15^ reported that only CMvD was a significant predictor for parafoveal scotoma in multivariate analyses, although BP measurements, such as office-hour MAP and OPP, showed significant association with parafoveal scotoma in univariate analyses. Considering the significant relationship between nighttime DBP dip and the presence of CMvD in the current study, we hypothesize that nocturnal hypotension may induce ischemia in the parapapillary choroid (e.g., CMvD). This, in turn contributes to the development of parafoveal scotoma, since CMvD is frequently observed at the inferotemporal region^12,15,16^, nearby the macular vulnerability zone, which is a narrow region of the ONH typically associated with parafoveal scotoma^40^.

Our study had several limitations. First, we had a relatively small sample size for the matched subgroup analysis (n = 60 in total). In the current study, subgroup analysis was performed for the eyes with early-to-moderate severity of VF damage, matched by VF MD (≤ 1 dB) and age (≤ 10 years), in eyes with and without CMvD, since the presence of CMvD is significantly related to the severity of glaucomatous damage and age^13,14^. The small number of patients in each group may also limit the generalizability of our findings to the general population with glaucoma. Second, although there was no significant difference in the proportion of antihypertensive medication use between groups with and without CMvD, information regarding different types of oral antihypertensive medications were not available, due to a lack of self-reported information from patients. Calcium channel blockers can induce a steal phenomenon and cause a sudden and extreme reduction of BP^41^, although there was no association between extreme nocturnal DBP dip and use of antihypertensive medication in our logistic regression analysis (Table 6). Further studies including large numbers of glaucoma patients, stratified by use of different types of oral antihypertensive agents, are needed to evaluate the impact of these agents on 24-h ABPM readings and its association with CMvD. Third, subjectivity can intervene in the determination of CMvD, although we attempted to minimize this by using 2 independent examiners and a 3rd adjudicator. In addition, eyes with partially reduced microvasculature may have been classified as not having CMvD, because we defined CMvD as a complete loss of the choroidal microvasculature, with a size of ≥ 200 μm in diameter. Fourth, projection artifacts or shadow effects may lead to under- or over-estimation of CMvD. Thus, our results should be interpreted with caution considering the technical limitation of OCT-A imaging. Quantitative measure of overall parapapillary choroidal vessel density, followed by examination of the relationship between 24-h ABPM and choroidal vessel density, may mitigate this issue^42^, and provide an alternative means to study the influence of BP dip on CMvD or parapapillary choroidal ischemia. All participants in the current study were Korean NTG patients with β-PPA, thus preventing generalization of our results to those without β-PPA or other types of glaucoma with different racial/ethnic groups. Various IOP-independent factors, such as vascular dysregulation or systemic hypotension, may be more closely associated with Koreans or the pathogenesis of NTG^43–45^. Selection bias may have been present as our patients were enrolled at a large university practice rather than in population-based settings; thus, they may not possess the same characteristics as similar patients in the general population. Finally, because of the cross-sectional study design, we could not assess the longitudinal relationship between various nocturnal BP dip parameters and CMvD detection. Longitudinal studies are required to evaluate the temporal relationship between nocturnal hypotension and the emergence of or change in CMvD.

In conclusion, NTG eyes with CMvD had nighttime DBP dip of greater magnitude and longer duration than eyes without CMvD. Moreover, nighttime DBP dip parameters (“time”, “area”, and “%”) were significantly associated with the presence of CMvD in NTG eyes. Despite that 24-h ABPM provides clinicians with valuable information regarding nighttime DBP dip and parapapillary/ONH ischemia, it may be too burdensome to perform 24-h ABPM in all glaucoma patients. Since our study demonstrates that the eyes with CMvD are closely associated with having nighttime DBP dip, NTG patients with CMvD should be recommended to undergo 24-h ABPM. Based on the results of 24-h ABPM, the modulation of nighttime DBP dips may prevent or slow down future glaucoma progression.

## Methods

### Study subjects

This cross-sectional study enrolled consecutive NTG patients from the cohort of an ongoing prospective study evaluating the relationship between NTG and systemic BP, which began recruitment from January 2013 at the glaucoma clinic of Asan Medical Center, Seoul, Korea. The study protocol was approved by the institutional review board of Asan Medical Center, and all procedures were carried out in accordance with the principles of the Declaration of Helsinki. Written informed consent was obtained from all participants.

All subjects underwent complete ophthalmologic examinations, including best-corrected visual acuity (BCVA), slit-lamp biomicroscopy, gonioscopy, Goldmann applanation tonometry, a manifest refraction test, AL (IOLMaster; Carl Zeiss Meditec, Dublin, CA) and CCT measurements (DGH-550; DGH Technology, Exton, PA), dilated color fundus photography, stereoscopic optic disc photography, red-free fundus photography (AFC-210; Nidek, Aichi, Japan), standard automated perimetry (Humphrey Field Analyzer with Swedish Interactive Threshold Algorithm standard 24-2 test; Carl Zeiss Meditec), cpRNFLT measurement using Cirrus HD spectral-domain optical coherence tomography (SD-OCT, Carl Zeiss Meditec), and OCT-A (Angiovue; Optovue Inc, Fremont, CA). SBP and DBP were measured once during office clinic hours.

NTG was defined as the presence of an open anterior chamber angle on gonioscopy, the absence of an identifiable secondary cause of glaucoma, a maximum bilateral untreated IOP < 22 mmHg in the outpatient clinic at 9:00 AM, 12:00 PM, and 4:00 PM, signs of glaucomatous optic neuropathy (i.e., vertical cup-to-disc [C/D] ratio > 0.7; asymmetry in the vertical C/D ratio between the eyes exceeding 0.2, and not explained by optic disc size; focal or generalized neuroretinal rim thinning; or RNFL defect), and compatible glaucomatous VF loss^9^. Eyes were considered to have glaucomatous VF loss if the glaucoma hemifield test results were outside normal limits and the PSD had a *P* value < 5%, confirmed on 2 consecutive reliable VF tests. When the first VF result showed glaucomatous defects, the first perimetric result was excluded from analysis, to obviate learning effects. All VF tests had to be reliable, defined as false-positive and false-negative error rates < 15% each, and fixation loss < 20%.

To be included in the current study, all NTG subjects had to meet the following criteria: no prior glaucoma treatment, age > 18 years, BCVA of 20/30 or better, a spherical equivalent between − 8.0 and + 3.0 diopters (D), cylinder correction within + 3 D, and visible β-PPA on fundus photography. All included participants underwent in-hospital 24-h monitoring of IOP and BP in the habitual position (sitting during the daytime [8 AM–10 PM] and supine at nighttime [12 AM–6 AM]). The affected eye was selected in patients with unilateral disease. If both eyes of a patient were eligible, 1 eye was selected at random.

Patients were excluded from the study if they had outpatient IOP > 21 mmHg; a history of intraocular or refractive surgery; pathologic myopia (patchy chorioretinal atrophy, lacquer crack, intrachoroidal cavitation, or choroidal neovascularization) or other evidence of retinal pathology that could affect the VF test; opaque media, such as visually significant cataract or BCVA < 20/30; previous or current use of systemic or topical steroid; and any history of neurologic or ophthalmic disease that could lead to VF abnormality^9^. Individuals who smoked or had irregular daily sleep patterns were also excluded. However, individuals taking systemic antihypertensive agents were not excluded^9^.

### In-hospital 24-h intraocular pressure and ambulatory blood pressure monitoring

The method of measuring in-hospital 24-h IOP an ABPM has been extensively described in our previous studies^9,10,46,47^. Briefly, all subjects were instructed to abstain from alcohol and caffeine for 3 days before hospital admission. IOP in both eyes of each patient was measured using the TonoPen XL (Mentor Ophthalmics, Santa Barbara, CA) every 2 h, from 8 AM to 10 PM, in a sitting position (daytime IOP) and every 3 h from 12 to 6 AM, in a supine position (nighttime IOP). This schedule was used to provide the best tradeoff between the maximal number of IOP readings over 24 h and minimal nonphysiological responses during in-hospital IOP measurement^9,10,46^. The IOP was measured 3 times in each eye, and average IOP was used for analysis. Various IOP parameters, including the mean, peak, and range of daytime and nighttime IOPs were calculated separately.

In-hospital 24-h SBP, DBP, and heart rate were measured every 30 min using a fully automated ABPM device (Spacelabs Healthcare, Issaquah, WA). This automated device minimized the variability among operators and measured BP in the most physiological environment, while patients continued their routine 24-h activities. The mean, peak, trough, and range of daytime and nighttime SBP and DBP were calculated separately, since short-term (daytime or nighttime) BP data and its variability over 24 h independently contribute to end-organ damage, including glaucoma, while concurrently avoiding the use of single daytime and nighttime BP readings^4–6,48–52^. Patients were encouraged to continue their normal indoor activities during the day and were asked to refrain from any physical activities that could affect BP at night.

### Nighttime and daytime blood pressure dip parameters

The magnitude and duration of the nighttime BP dip relative to the mean daytime BP level (i.e., nocturnal BP trough > 10 mmHg below the average daytime BP) is predictive of future glaucoma progression^7,9,10^. Therefore, we investigated the magnitude and duration of the nighttime and daytime BP dip relative to the mean daytime BP value, using the following 3 variables: (1) “%”, (2) “time”, and (3) “area.”

The “%” of the nighttime and daytime BP dip was calculated as^9,10^:

$$ \begin{gathered} {\text{Nighttime BP dip }} (\%) \, = \,\left( {{\text{mean daytime BP}}-{\text{trough nighttime BP}}} \right)/{\text{mean daytime BP}}\, \times \,100. \hfill \\ {\text{Daytime BP dip }}(\%)  = \,\left( {{\text{mean daytime BP}}-{\text{trough daytime BP}}} \right)/{\text{mean daytime BP}}\, \times \,100. \hfill \\ \end{gathered} $$

The “time” (in h) of the nighttime and daytime BP dip was defined as the total time spent at least 10 mmHg below the mean daytime BP during nighttime and daytime, respectively^7,9^. The “area” (mmHg h) of the nighttime and daytime BP dip was defined as the time multiplied by the magnitude of the nighttime and daytime BP that was at least 10 mmHg below the mean daytime BP, respectively^7,9^. In the current study, the “%”, “time”, and “area” of the SBP and DBP dip during daytime and nighttime were calculated separately. Finally, the extreme nocturnal BP dip was defined as an abnormal decrease in the nighttime BP trough exceeding 20% in relation to the mean daytime BP^10^.

### Choroidal microvasculature dropout and circumpapillary vessel density measurement using optical coherence tomography angiography

The AngioVue OCT-A imaging system (Software version 2017.1.0.144; Optovue Inc.) facilitates noninvasive visualization of the ophthalmic microvasculature. This OCT-A system has a light source with a center wavelength of 840 nm, a scanning speed of 70,000 A-scans per second, an axial resolution of 5 μm, and a transverse resolution of 15 μm. A split-spectrum amplitude-decorrelation angiography algorithm was used to identify perfused vessels by capturing the dynamic motion of moving particles, such as red blood cells (RBCs)^53^. All NTG participants underwent OCT-A imaging of a 4.5 × 4.5 mm^2^ region centered on the optic disc. Only qualified images with a signal strength index (SSI) > 45, no motion artifacts, and segmentation error were analysed.

The choroidal microvasculature was evaluated within the β-PPA area, based on the choroidal layer of the OCT-A image. The β-PPA was defined as an atrophic region of the retinal pigment epithelium resulting in a distinctive appearance of scleral and large choroidal vessels^54^. The β-PPA area was outlined and calculated on scanning laser ophthalmoscopic images by 2 observers (J.W.S. and Y.J.) using ImageJ software (version 1.51; National Institutes of Health, Bethesda, MD, USA). The Littmann formula was used for correction of AL-related ocular magnification effects in measuring the β-PPA area^55,56^. Average values of the β-PPA area measurements from the 2 examiners were used in analyses. The CMvD within the β-PPA was defined as a complete loss of the choriocapillaris and choroidal microvasculature, without any visible microvasculature network, as defined in previous studies^12,13^. The minimum width of the CMvD at the smallest part of the lesion was required to be 200 μm or greater than the width of the central retinal vein, to avoid false positive findings^13,16,57^. 2 observers (J.W.S. and Y.J.) independently determined the presence of CMvD, blinded to the clinical information of participants. Disagreements between these 2 observers were resolved by a third adjudicator (M.S.K.).

The cpVD was evaluated within the radial peripapillary capillary slab, from the internal limiting membrane to the nerve fibre layer. The cpVD was calculated as the percentage of measured area occupied by vessels with flowing RBCs in a region defined as a 1000-µm-wide elliptical annulus extending from the optic disc boundary.

### Spectral-domain optical coherence tomography evaluation

Optic disc cube scans, obtained using the Cirrus HD-OCT (software version 10.0), measured RNFL thickness at peripapillary regions (6 × 6 mm^2^) centered on the optic disc. The average cpRNFLT was measured in a 3.46-mm diameter circle. Poor quality SD-OCT images, with motion artifacts, segmentation failure, or signal strength < 7, were excluded.

### Statistical analysis

Interobserver agreement regarding the presence of CMvD was calculated by kappa statistics. The interobserver reproducibility for β-PPA area measurement was assessed by calculating the ICC. Clinical characteristics and in-hospital 24-h IOP and ABPM parameters were compared between eyes with and without CMvD. Continuous variables were compared using independent Student’s *t*-tests or Mann–Whitney *U* tests, depending on the result of the normality test (Shapiro–Wilk test). Categorical variables were compared using the chi-square test. Univariate logistic regression analysis was performed to determine potential clinical variables (i.e., office BPs, in-hospital daytime and nighttime SBP, DBP dip, and IOP parameters) associated with the presence of the CMvD. A backward elimination process was used to build a multivariate logistic regression model incorporating variables with P < 0.10 in univariate analysis. Multicollinearity among independent variables in the multivariate model was assessed by quantifying the variance inflation factor (VIF), with high multicollinearity defined as a VIF > 10^58^.

Subgroup analysis was performed for eyes with early-to-moderate severity of VF damage (MD > -12 dB). Subgroups were matched by VF MD (≤ 1 dB) in eyes with and without CMvD, since the presence of CMvD is significantly related to the severity of glaucomatous damage^13,14^. In addition, 2 subgroups with and without CMvD were also matched by patient age (≤ 10 years). Finally, the factors associated with extreme nocturnal DBP dip were also determined using logistic regression analyses, as a profound DBP dip is significantly linked to ONH ischemia and NTG^3,9^. *P* values < 0.05 (two-tailed) were considered statistically significant. All statistical analyses were performed using SPSS software (ver. 20.0; IBM Corp., Armonk, NY) and the R statistical computing package (ver. 3.1.2; R Foundation, Vienna, Austria).
